# Promoting Coevolution Between Healthcare Organizations and Communities as Part of Social and Health Pathways Management in Quebec: Contributions of the Complex Adaptive Systems Approach

**DOI:** 10.1177/11786329251332797

**Published:** 2025-04-18

**Authors:** Lara Maillet, Georges-Charles Thiebaut, Anna Goudet, Jean-Sébastien Marchand

**Affiliations:** 1 National School of Public Administration (ENAP), Montreal, QC, Canada; 2 Institut Universitaire de première ligne en santè et services sociaux (IUPLSSS), Sherbrooke, QC, Canada; 3 Centre de recherche Charles-Le Moyne (CRCLM), Longueuil, QC, CANADA; 4 Canada Research Chair in Adaptive Systems for Health and Social Services Tier 2, CRC Sa3S, Laboratory for Systemic Health Research and Interventions (LabRIS), ENAP, Montreal, QC, Canada; 5 Commissaire à la santé et au bien-être/Commissionner of Health and Wellness (CSBE), Montreal, QC, Canada

**Keywords:** coevolution, health care systems, health services management, healthcare equity, healthcare governance, complex adaptive system

## Abstract

The implementation of sociosanitary pathways in the Quebec healthcare system aims to better meet the needs of communities and strengthen their participation at all levels of governance. This initiative will form the basis of our article, which will look at the challenges of adaptation both inside and outside organizations. Drawing on the complex adaptive systems approach, we have developed an analytical framework to highlight the processes that can lead to the adaptation of governance to facilitate community participation in the management of this pathways. The aim of this article is to propose a better understanding of coevolution in the process(es) of adaption of the governance of a complex healthcare organization to its environment, by mobilizing the complex adaptive systems approach. We conducted a qualitative case study, based on 4 sources: documents (n = 70) produced or used during implementation, participatory observations on various tactical and operational committees of the management structure, collaborative workshops with members of the management committee, and semi-structured interviews (n = 18) with managers, department heads, partners, and users of health and social services. To understand the co-evolutionary processes involved in the implementation of management by social and health pathways, we present our results in response to 3 research proposals on the theme of internal and external coherence in a healthcare organization, in terms of vision (cultural), structures (organizational and clinical), and relationships with external partners (environment). Our findings show that to implement and manage an innovation in a healthcare organization, it is fundamental to foster coevolution at operational, tactical and strategic levels, as well as with the external environment. To achieve this, it is necessary to maintain a balance and internal coherence between the structure being implemented and the existing structure, to establish formal and informal communication channels to ensure seamless interactions, while recognizing and reinforcing mutual interdependence in a systemic perspective.

## Introduction

In 2015, the Quebec government undertook a major reorganization of its health and social services network. The main objectives of this reform were to promote and simplify access to services for the population, contribute to improving the quality and safety of care, and increase the efficiency and effectiveness of this network.^1^ The *Act to Amend the Organization and Governance of the Health and Social Services Network* (Bill 10) brought about major changes in the governance and organization of healthcare organizations, including major mergers and a significant centralization of power to the Ministry of Health and Social Services.^2^

As part of this province-wide structural reorganization, several healthcare organizations have developed social and health pathways, and a management model to support them: social and health pathways management.

The implementation of social and health pathways represents a governance innovation that aims to increase the abilities of health and social service organizations to adapt to the needs of the population, particularly through the improvement of accessibility and continuity of services.

This innovation has 2 main elements. Firstly, the implementation procedure is based on the strategic community approach.^3,4^ Strategic communities are characterized by inter-organizational collaboration structures consists of professionals, first-level executives, general practitioners, specialist physicians, and representatives of community organizations whose mandate consists of generating, putting into practice and assessing new ideas regarding the organization of work between establishments.^3^ This means that the implementation of social and health pathways is supposed to be based on collaboration and cooperation between all actors involved in a social and health pathway in which both users and community partners are heavily involved. The method of operation should therefore no longer be hierarchical, but cooperative, with a large part left to the emergence and co-construction of diagnoses and solutions.^4^

Secondly, this innovation seeks to introduce a matrix structure to the organization in order to make a connection between the implementation structure and the management structure.^5^ Those who are enforcing the implementation process are functional specialists who are responsible for the application, operation, and improvement of social and health pathways through the creation of new methods for the organization of services.^6^ Coordination along the horizontal line of the matrix (the social and health pathways) and between the horizontal and vertical line (the management systems) should be achieved through mutual adjustment between the administrative and clinical professionals at all levels of governance.^6^ This type of structure aims to increase the flexibility and the transversality of the organization so that it can adapt appropriately to meet the needs of the environment (users, partners, population of the area).

### Complex Adaptative System (CAS)

The innovative nature of this governance implies a global and systemic vision of this implementation. Building on previous work,^7,8^ we have developed a systemic analysis framework based on complexity.

**Complex Adaptive System (CAS)** has 3 main characteristics: many actors or components; they are different and autonomous; and, above all, they are interdependent.^9–11^ Their interdependence means that the influence of 1 actor or component is linked to the presence and intensity of other actors or components.^12–14^ This relationship is not linear. It is curvilinear. It is therefore through the interactions between these actors (or components) that this interdependence reveals its presence.^15^

**Figure 1.**
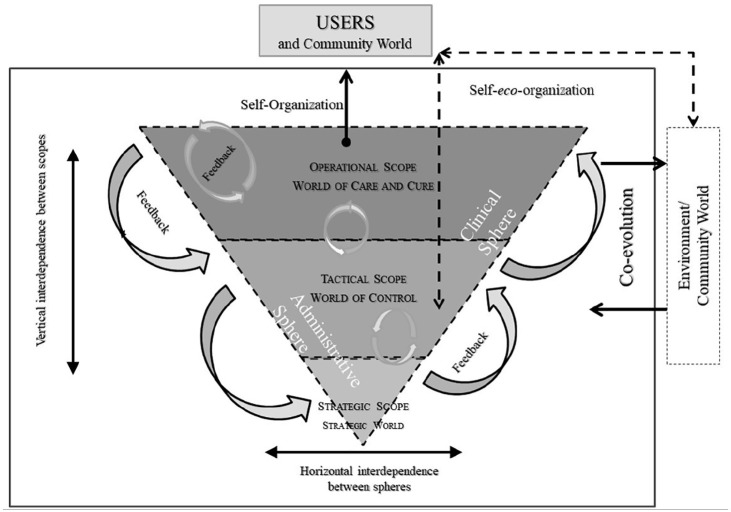
Representation of multilevel governance in a complex health care organization. Source: Adapted from Maillet et al.^7^

Jessop^16^ describes governance as a complex art, and multilevel governance allows us to describe new forms of public authority at various levels: local, national, and supranational. From this perspective, the use of multi-level governance can be encouraged by the combination of operational autonomy and interdependence between organizations and systems.^7,17^ This use is considered in a pluralistic context, in which it is impossible to conceive of any absolute authority due to shared leadership and diffuse powers.^18,19^ In a healthcare context, drawing on the work of Folke et al.,^20^ Lamothe,^21^ and Maillet et al.,^7^ multilevel is structured across 3 levels (operational, tactical, strategic): the operational corresponds to the frontline clinical-administrative sphere, the tactical to the management layer, and the strategic to the “hierarchical” decision-making tier.^6,22^ From this perspective, governance serves as a space where actors at the operational level benefit from the credibility associated with the tactical and strategic levels. Conversely, administrators of the strategic level require an ongoing legitimacy from the other levels, particularly from healthcare professionals. The tactical level plays a crucial role by translating strategic guidelines to the operational level and conveying actions undertaken at the operational level up to the strategic level.^23^ This structure fosters coherence across the 3 levels, strengthening the processes of adaptation and organization. Healthcare organizations are thus thought of in terms of interaction and co-evolution between stakeholders, going beyond a purely hierarchical vision.^24^ (Figure 1).

Environment is defined as all the players around healthcare organizations. These may be community organizations, government departments, the community, etc. The Environment can be local or more global. By interacting with its local and global Environment, the organization shapes itself by taking it into account and learning from it (ie, its context of action involving community representatives, municipalities and all players external to the healthcare organization).^20,25^

**Co-evolution** refers to the iterative process whereby “organizations attempt to meet the expectations of their environment, and their environment changes according to the efforts made by the organizations and the expectations they succeed in meeting. This process implies that organizations create their environment, which in turn shapes the organizations” (p. 58).^26^ This is also known as the interdependence of organizations and environments.^27^ They are continuously connected and dependent on each other through positive feedback processes.^15^ The organization and its environment are mutually adaptive.^28^

Finally, **coherence** refers to the harmony, logic or fit between different parts of a whole. In the context of management, coherence can be defined as the ability to maintain alignment between an organization’s strategies, objectives, actions, and values. This ensures the effectiveness and relevance of the decisions taken. To achieve this, decisions must be taken in line with organizational priorities and the priorities of the external parties with whom the organization is in contact, that is, the environment. In a complex system, interdependence is continuous and dynamic; coevolution is the result of this dynamic interdependence.^29^

### Social and Health Pathway Management Structure in Healthcare Organization A

In Quebec, the notion of pathways is quite broad. It integrates the social and health dimensions, is user-centered and conceived as an interweaving of several episodes of care and services over long periods of time in an intersectoral perspective in a targeted territory. It brings together users with a similar clinical condition or profile and can extend over the whole of a person’s life by integrating access mechanisms, health promotion, prevention, treatment, follow-up, and end-to-life care.

This article focuses on the implementation of social and health pathways management in 1 case, the Healthcare Organization A. This empirical case study is rich in lessons, particularly because of its duration and scope. Unlike several other organizations, which did not go beyond the experimentation or pilot project phase, the Healthcare Organizations A deployed social and health pathways management throughout the organization and across the entire regional territory.

The Healthcare Organizations A is a large-scale regional organization that provides almost all the healthcare and social services for the population of its region. Its services cover all stages of life, from birth to end-of-life care, and include preventive, specialized (such as surgery, oncology, and radiology) as well as subspecialized (such as neurosurgery and neonatology) care. Created by the merger of the territory’s various healthcare establishments in April 2015, the Healthcare Organizations A serves around 500,000 inhabitants spread over an area of 13,000 km² and has around 20,000 employees working in more than 100 facilities organized into 9 local service networks.

In this organization, the desire to implement social and health pathways comes directly from the impetus of the executive management from 2016. A process management approach is adopted for the implementation of social and health pathways, inspired by Lean management. The implementation approach makes the users the central focus, and advocates the participation of all actors (clinic, administration, and community/intersectoral partners) in the spirit of shared responsibilities.

Social and health pathways is implemented by a **Social and health pathways operational committee (SHP-OC)** that brings together the clinical and administrative co-owners (physicians and management), clinicians, members of the various departments concerned, community partners, and users. **SHP-OC** contributes to the deployment and ensures the supervision of the care pathways. It identifies and commissions the necessary workgroups in order to make improvements according to a fixed deadline and ensures their support.

**Figure 2.**
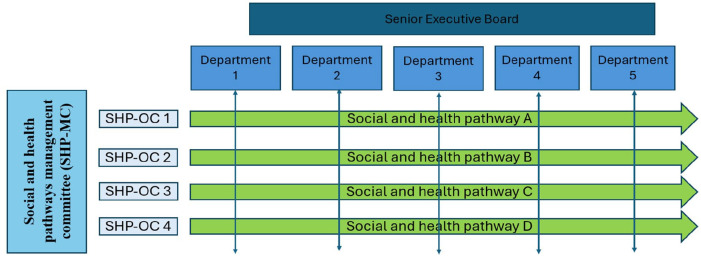
Social and health pathways management structure illustration in Healthcare Organization A.

A **Social and health pathways management committee (SHP-MC)** supports all SHP-OC. It contributes to the deployment of the approach and to the creation, running, and evaluation of the stages of the implementation process and the tools used; documents the results, and generates, shares, and consolidates findings; oversees managerial intercommunication and reports organizational governance issues (Figure 2).

The aim of this article is to propose a better understanding of coevolution in the process(es) of adaption of the governance of a complex healthcare organization to its environment, by mobilizing the complex adaptive systems approach.

## Method

### Design Study

As part of this study, we conducted a qualitative case study with nested levels of analysis^30^ over an 18-month period (2017-2019). The aim was to better understand how collaborative dynamics developed between the SHP-MC and SHP-OC committees, as well as the Executive Board as part of the implementation of social and health pathways management within a healthcare organization (case A). We were able to observe the iterative design cycles that led to the formalization of structures, management processes, and social and health pathways within the healthcare organization and with the network of partners and users involved on different SHP-OC. We were able to document the interdependence and coevolution within these different processes.

### Data Collection

Data were collected from 4 sources (Table 1): documents produced or used during implementation, participatory observations on various tactical and operational committees of the management structure (SHP-MC, caucus and SHP-OC), collaborative workshops with members of the SHP-MC, and individual semi-structured interviews with managers, department heads, partners, and users of health and social services. A total of 70 documents relating to the pathways structure and process were assembled and analyzed (meeting reports, pathways planning and management tools, monitoring indicators, etc.).

**Table 1.** Data collection.

Documentary analysis	Participatory observation (SHP-MC and SHP-OC)	Collaborative workshops	Semi-directed individual interviews
70 documents	32 committees 200 h	3	18

Participatory observations were documented using observation grids between January 2017 and June 2018 (n = 32 committees observed or over 200 hours of observations). Data and documents from collaborative workshops, held as part of the developmental evaluation, with SHP-MC members and the research team were also collected and analyzed (n = 3 workshops). The purpose of these workshops was to provide reflective feedback on practical issues related to the development and implementation of social and health pathways management and linking them to evidence from the scientific literature or international experience.

Finally, we conducted 18 individual semi-structured interviews between May and August 2018. Participants for the interviews were recruited internally by purposive sampling (see supplemental material here). The inclusion criteria were to be part of one of the different committees of pathways management: SHP-MC, SHP-OC, and caucus. The administrative and clinical spheres separate the participants according to whether they belong to the world of management and administration or the world of social-health practice (healthcare professionals and social workers).

They were proposed from key collaborator, then they were approached by email by the researchers to find out if they were interested in participating in the research. None refused or dropped out. The representativeness of the interviews is strong because a high number of managers and stakeholders from different spheres, sites, and fields were involved in the project.

The distribution of participants in these interviews is presented in Table 2. Recruitment by purposive sampling also respected the criterion of relevance, particularly with partners and users. The sample size was not calculated a priori; we proceeded by reaching a saturation point.^31^ Each interview began with the explanation and signature of the consent form approved by the Ethics Committee.

**Table 2.** Distribution of interviews by sites, action levels, and spheres.

Sites	Strategic		Tactic		Operational	
	Administrative sphere	Clinical sphere	Administrative sphere	Clinical sphere	Administrative sphere	Clinical sphere
Organization	2	1	3	1	4	12
Partners	3					3
Users					4	4
Total	5	1	3	1	8	18

Interviews were recorded, with consent being sought from those involved. Some interviews took place in person at the workplace, others virtually. They were conducted by the researchers responsible (first and second authors), and research assistants. The interview guide was tested and adapted during the initial interviews. Questions included for example: “Who are the main players involved in pathways management? What do you think of the collaborative links between the various stakeholders involved? What facilitates and/or hinders this collaboration? Could you tell me how and by whom you learned about pathways management? What was your role?” Interviews lasted between 1 hour and 1 hour 30 minutes.

### Data Analysis

Based on Miles and Huberman’s framework of qualitative methodology,^32^ the analysis and collection was carried out simultaneously and iteratively in terms of coding and categorization, which enabled the researchers team to adjust the interview guides and observation grids.

To capture the data and facilitate analysis, QDA Miner software version 5^33^ was used. The first phase of analysis involved listing the units of meaning corresponding to the codes (descriptive, interpretive, and explanatory) and characterizing basic concepts to highlight emerging themes, configurations, and patterns of explanation. The codebook was used to assemble data from documents, observations, and interviews into a corpus relevant to our research objectives. The main categories of the codebook are: Respondent characteristics, Initial implementation conditions, Innovation development, Implementation process, Governance structure, User and partner participation, Outcomes and perspectives.

To ensure the triangulation of data from several respondents and several instruments (observations and documentary sources), we had to control the fidelity and validity of our analyses. The main reseearcher (first author) and a research assistant carried out “coding-contrecoding” to ensure that the transformation, synthesis, and reduction inflicted on the data corpus remained faithful and valid to the raw data. It should be remembered that codes and themes were kept for analysis as long as 3 respondents, regardless of organization or field of affiliation, brought them to light. This was valid for codes pre-established from the theoretical framework and the literature, or for codes emerging directly from the corpus of data as the analysis progressed.

A second phase involved mapping the dimensions of collaborative dynamics (eg, joint action capabilities) for each site, governance level, and sphere. The data were reduced (matrices, relationship mapping, case memos, and summaries), enabling our research propositions to be analyzed on the basis of the data collected.^32^.

### Ethical Process and Consent to Participate

Research Ethics Committee of the Centre intégré universitaire en santé et services sociaux Estrie-CHUS approved pilot studies (#MP-31-2018-2784) and the principal project (#MP-31-2021-3799). All participants provided written informed consent prior to participating.

## Results

To understand the role played by the coevolution in the process(es) of adapting the governance of a complex health organization to its environment, we will present our results in response to 3 research propositions on the theme of internal and external coherence in a healthcare organization, in terms of vision (cultural), structures (organizational and clinical) and relations with external partners (environment). These research proposals are working hypotheses that we wanted to validate using the data collected and the analyses carried out. In addition to the data collected, we validated the results with resource persons within organization A in order to fully grasp the particularities of each of the levels analyzed.

### 1/ The Internal Coherence of the Implementation Structure and Process Depends on the Level of Understanding and Appropriation of a Common, Shared Vision of the Structure of Implementation of Social and Health Pathways Linked with the Role of the Involved Users and Communities’ Partners

**Figure 3.**
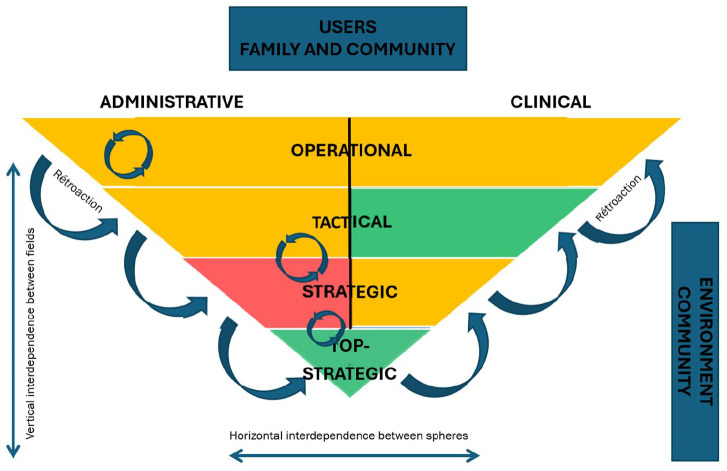
Cross-analysis of internal coherence (research proposition 1).

In the context of implementing social and health pathways within healthcare organizations, the internal coherence of the implementation process is critically dependent on how well a common vision is understood and embraced across different organizational levels. A shared vision acts as a unifying framework that guides the coordination and management of pathways, ensuring that all stakeholders are aligned in their goals and approaches. The challenge lies not only in developing this vision but also in effectively disseminating and embedding it throughout the organization (Figure 3).

**At the strategic level** of Healthcare Organization A, senior management clearly articulates the vision for the implementation of social and health pathways.

“*The Pathway must remain a priority in the minds of directors, and they must be committed to implementing the solutions arising from the trajectories. [. . .] Pathway is not a project, it’s a way of doing things on a daily basis. The project must become the organization’s identity*.” (Observation notes, Extract from senior management’s opening remarks at the SHP-MC meeting, November 22, 2017).

However, this clarity does not always permeate down to the directors and deputy directors of clinical and support divisions. For instance, although senior management communicates the importance of these pathways, this topic does not consistently appear as a recurring agenda item in the Management Committee meetings, which are critical forums that bring together all departments within the institution. There is also an absence of strategic committees dedicated to the discussion and management of social and health pathways, leading to fragmented communication and inconsistent understanding across the organization.

“It hasn’t belonged since the beginning, so it hasn’t been carried anywhere. It’s supported in terms of vision, but not action. How can I tell you this? [. . .] We’re all SHP-OC managers and we belong to the departments. There’s no superior managing this.” It needs stronger leadership at times. (Respondent #2)

In addition, there is ambiguity as to the degree of priority of the pathways and the coherence between the various “innovations,” particularly the one on performance. A respondent related:

“OK, I’ll share my opinion on this political issue. When the government announced the mergers in 2015, the creation of huge structures, I remember senior management saying they’d make it better. The customer is on the motorway. When you need petrol, you stop at a station and continue. All the programmes/services are defined as partner stations. The new structure would do this. No more silos. It was beautiful! I even thought, ‘Maybe reform is a good thing.’ Three years on and we’re still in the same situation. We’ve never been in silos before, because it’s all getting mixed up. With the performance context, we’re moving towards performance evaluation for performance-based financing . . . Our access times, client numbers, interventions per day, etc. are used to determine our performance and funding. If we’re underperforming, we’ll be cut. We’ll argue about the petrol station closing and the client taking up time and using up petrol. That’s incompatible. I’m all for health pathways, but we’re doing this in the context of a health organisation, disregarding local practice, and that’s very worrying.” (Respondent #22)

Finally, there is a certain variability in the understanding of their role by administrative and medical co-owners in the management of pathways. This situation creates complex tensions, reflecting varying levels of commitment and engagement among different healthcare organizations’ stakeholders, thereby undermining the coherence of the implementation process.

“18 months after the start of work on the SHP, the actors at the meeting made their mea culpa to the pathway co-owners for the little or no involvement they had been asked to provide. They underline the difficulty with the mandate expected of co-owners: Role? The term “co-owner”: difficulty when you don’t control anything. Accountability for what? Collaboration? In what and how?” (Observation notes, SHP-MC meeting, June 6, 2018)

One respondent confirmed this gap in an interview:

“*We probably should have involved the co-owners more in the development of the project, so that their vision would also be reflected and create an ownership of their role on an ongoing basis, and so that there would never be a gap between what we do and what they must assume as their role*.” (Respondent #1)

**At the tactical level**, there is a strong alignment in the understanding of the coordination processes for social and health pathways, particularly among those responsible for these pathways. These individuals are often closely involved in the co-development and piloting of pathways, which facilitates a shared vision within their immediate teams. However, divergences arise when it comes to the implementation and outcomes of these pathways. For example, there are differences in how pathway objectives – such as stabilizing service offerings, ensuring continuous improvement, and enhancing accessibility and fluidity – are prioritized and interpreted. A respondent highlighted:

The working group was making progress, yet others felt excluded from development. There are challenges, but we worked with 19 people. I think the foundations were laid, with good representatives from everywhere. People are open to suggestions. We reevaluated because the [SHP-MC] changed, there were sub-workgroups and we changed the interlocutor to a group. We used the same pathway management mechanism, with an executive preparing the [SHP-MC] meetings and acting as intermediary with top management. We assessed what the executive had done, but also where we were and what our game plan was. We’d achieved our goals: launched pathways; conducted SHP-MC meetings; discussed indicators; tackled bottlenecks; and reviewed our SHP-OCs. “We tracked the number of SHP-OCs and their location. We followed the two pilot pathways, following the stages.” (Respondent #20)

This variability in interpretation highlights the challenges of maintaining internal coherence, especially when the vision is not uniformly internalized or when the roles of various stakeholders are ambiguously defined.

**At the operational level**, the implementation of SHP-OC has successfully fostered collaboration and the development of a shared vision among participating actors, including clinical staff, users, and community partners. These efforts have helped bridge gaps between different stakeholders, aligning their goals and facilitating smoother implementation of pathways. However, outside these structured interactions, there remains a low level of awareness regarding the principles of pathway coordination and the concept of pathways:

“For me, it was an irritant: we’re going to export our model [as part of the ministerial committee], when we haven’t even explained it internally. For me, one of the things that would have made it easier would have been to say, this is where we want to go, well, we’re having a senior management forum and we’re talking about all this, so at the same time, the whole establishment community knows where we’re going and what’s expected.” (Respondent.e #2)

This limited understanding at the operational level further exacerbates the challenges of maintaining internal coherence across the organization.

To support a shared vision and enhance internal coherence, it is essential to address several cross-cutting issues. At the strategic level, there needs to be a clear prioritization and communication of the innovation within the healthcare organization, utilizing multiple strategies for dissemination and appropriation. Ensuring alignment and coordination between management and operational structures is crucial:

“Sometimes, they give you an order at the bottom, which makes it top-down, and here we’ve got stuff here that we say “eh, it would be fun if we could . . .” but there’s no link between the two yet. (. . .) My budget is for the year. I don’t have the money to solve this problem. (. . .) And here at the top, when we’re working on this, we say that’s the priorities, that’s it. So it’s a new priority, a new problem, it’s not one of those at the top, it’s not budgeted at the bottom. So we have a financial problem to solve.” (Respondent #18)

The ambiguity surrounding the roles of co-owners further complicates the implementation. At the tactic level, while SHP-OC managers often play dual roles as both designers and champions of the innovation, this can lead to rapid development but also risks the innovation being too narrowly focused. Clearer delineation of roles and responsibilities, particularly among support directors, and increased involvement of SHP-OC managers are needed to enhance the internal coherence of the implementation process.

### 2/ Coherence Between the Implementation Structure and the Management Structure Facilitates the Implementation, Operation, and Continuous Improvement of Social and Health Pathways, as Well as the Participation of Users and Communities

The successful implementation, operation, and continuous improvement of social and health pathways within healthcare organizations depend heavily on the coherence between the implementation structure and the management structure. This coherence ensures that decision-making processes, resource allocation, and the flow and processing of information are aligned with the overarching goals of the organization, thereby supporting the effective participation of users and communities in these pathways.

**Figure 4.**
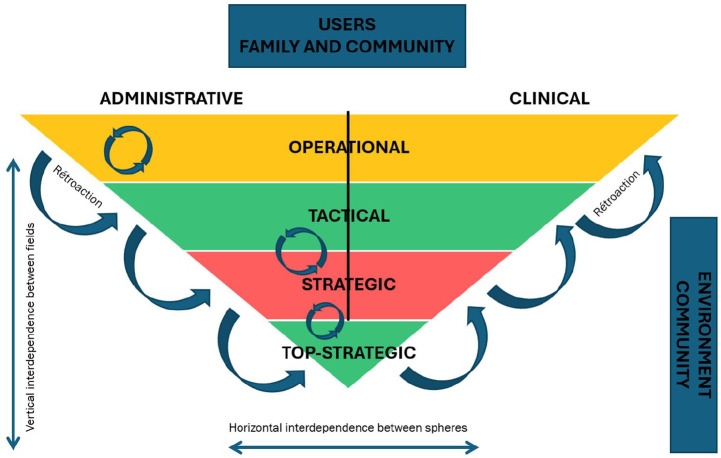
Cross-analyses of coherence between structures and action logic (research proposition 2).

Achieving coherence between implementation logic and management logic requires a strategic alignment of decision-making mechanisms across all levels of the organization. This alignment is crucial because it directly influences how resources are allocated and reconfigured in response to organizational priorities and efficiency criteria. Social and health pathways are not merely projects to be implemented; they represent essential mechanisms for improving population health. Therefore, it is critical to ensure that the decision-making structures and processes support these pathways effectively, from strategic planning through to operational execution (Figure 4).

Several issues arise when attempting to establish this coherence in Healthcare Organization A. A fundamental question is how to create a connection between the management structure and the implementation of social and health pathways. This involves understanding where decision-making occurs versus where it ideally should occur. For instance, while managers at the strategic level may allocate professionals to operationalize pathways, these roles are often not hierarchical, leading to ambiguities in decision-making and responsibility. In this regard, a respondent raises:

“Where I think [the SHP manager] is a bit stuck at the moment, as much as she wants to support her strategic role, there’s no body to take her issues [to the top]. Pathways are nowhere discussed at the strategic level. That’s why we need our model of management structure that’s going to provide some governance for it.” (Respondent #1)

This issue is compounded at the tactical level, where local managers face resource constraints that make it challenging to balance the demands of implementing cross-functional pathways with those of their clinical management duties in a vertical structure:

“As far as I’m concerned, I’ve got a lot of other things on my plate in addition to the “Health Pathways management”. We were supposed to have three days dedicated to this . . . That hasn’t been the case in the last two years. This year, I did what I had to do to reserve my agenda, because otherwise it’s done in the evening, it’s done elsewhere. You can’t turn up in front of 25 people and not be ready.” (Respondent #2)

The tension between hierarchical and functional structures is particularly evident in the governance of the healthcare organization. There is a lack of coordinated governance structures and processes at the strategic and tactical levels, which creates significant challenges. Without clear instances or processes for piloting and liaising at these levels, there is a risk of misalignment between strategic objectives and the deployment of social and health pathways. This misalignment can lead to issues with visibility and ownership at the operational level, particularly when parallel initiatives, such as Integrated performance management system^34^ (SIGP), dominate attention and resources. A respondent underlined:

“If we don’t move quickly, so that by fall we have a place where these decisions can be made, and there are big alignments coming down, we won’t be able to move forward (. . .) and when it doesn’t move forward it’s because it hasn’t been prioritized in their directions. And that’s all I can do. I can’t give the order for it to be prioritized. So that’s it . . .” (Respondent #1)

Decision-making and relevance are further challenged by issues related to data availability and utilization. In many cases, critical data – such as population and territorial data, as well as scientific knowledge – are either missing or not adequately integrated into decision-making processes.

*“Here, we can output our data, but it’s integrated into a data list, so . . . and it’s not an Excel format that we can go and sort, so we have to take the data, go and transcribe it into a file, contact the local establishments and all the other centers that do it, and ask for the same data. The examinations are not necessarily done in the same way, so we’ll have to see how . . ..” (Respondent #4)*

This gap makes it difficult to tailor the implementation of social and health pathways to the specific needs of different territories, leading to potential inefficiencies, and reduced effectiveness. Additionally, the absence of a common model for harmonizing and adapting analytical processes and tools across different pathways exacerbates these challenges:

“*We do evaluations, so I named this element. Of course, the impression I get is that there’s some analytical work done beforehand, and we’re made to wear a pair of glasses with . . . we extract data and we’re told, look, put on these glasses and make choices based on the data we’ve extracted. But we don’t know the analysis process that led them to extract the data. So I have the impression that we’re cut off from certain elements that could change the direction of a boat; you understand?*” (Respondent #27)

However, a shared vision focused on continuous improvement of social and health pathways (one of the four guiding principles of SHP management) goes some way to fostering commitment and consistency in the process:

“*I’d say we’ve managed to keep the momentum going, but that’s because we’ve developed a common vision, major principles, we’ve continued to feed, I don’t know if you’ve noticed, but we’ve had a lot of guests come in to talk about information management, the project, the ministerial mandate on this, to talk about . . . (. . .) so we continued, I think, to keep the mobilization going*.” (Respondent #1)

But it should be noted that the focus on accessibility and fluidity often comes at the expense of other important dimensions, such as relevance and efficiency, which are essential for the long-term success of the pathways. Fragmenting the service offering and the organizational structure does not make it possible to grasp complexity, which ultimately cannot be achieved in silos.

In conclusion, coherence between the implementation and management structures within healthcare organizations is vital for the effective implementation and continuous improvement of social and health pathways. Achieving this coherence requires strategic alignment of decision-making processes, resource allocation, and the flow of information. Without it, the potential benefits of these pathways – such as improved population health and enhanced participation of users and communities – may not be fully realized.

### 3/ External Coherence Between the Structure of Implementation of Social and Health Pathways, the Environment (Users, Partners, and Communities), and the Participation of Users Depends on a Clear, Shared Vision of Roles and Responsibilities

**Figure 5.**
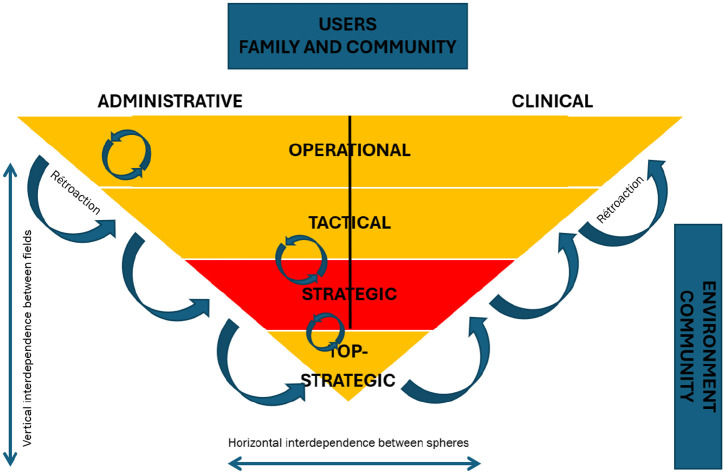
Cross-analyses of external coherence (research proposition 3).

For the effective implementation of social and health pathways, external coherence between the organizational structure and the environment – comprising users, partners, and communities – is critical. This external coherence hinges on a shared understanding and clear articulation of roles and responsibilities among all stakeholders. When such a vision is established, it facilitates the seamless exchange of information and coordination of processes between various actors, which is essential for the successful operation of user-centered pathways (Figure 5).

One of the main challenges highlighted here is how external perspectives, including those of users and intersectoral partners, are integrated into the healthcare organization’s approach to pathway implementation. At the operational level, the involvement of these external actors in SHP-OC activities has been a notable success, contributing significantly to the implementation of user-centered pathways.

« *Well, they thought it was . . . in any case, that’s what I like when I go there, is that the participating patients, we’re not put on the spot, but . . . we’re stars. I: [laughs] P: That’s how I see it. And what we say is taken down, it’s important, it’s not just “okay, next . . .”. They appreciate what we say, they’ll question it if our intervention isn’t clear . . . I’ve realized that it’s important and that it’s taken seriously and that it fits . . . it’s not “ok we write it down and forget it*”.

(Respondent #6 user)

« *I’m someone who takes her place very well too. I’m not afraid to say that I work in community organizations. We left a lot of room for parents too. They didn’t immediately want to raise their hand to speak, but we asked their opinion: “And you, do you think this kind of thing is feasible? Do you think . . .”, and then they’d open up. We gave them the chance to talk. I liked that*. » (Respondent #29 partner)

This involvement ensures that the pathways are grounded in the real needs and experiences of the users they are designed to serve:

People listen to each other. When the doctor heard the user, he said yes and agreed that it’s true and said he hadn’t thought that, but that he often hears it said by him. Psychosocial carers were also involved. They spent time organising things and collected a lot of irritants, but it was the users who made changes and showed what was needed. That’s how it rallies people around the real needs of the user. (Respondent #1)

It should be noted, however, that while the point of view of users and community partners is considered, they are not in a position of shared leadership either. Our observation notes underline this issue of the (real) role of user partners in governance:

“Most members comment that the room is impossible to find. [The user] comments that it is very difficult for him to access - he has physical functional limitations, and the room is far from the arrival of the adapted transport. No follow-up is done.

[The CTO manager] welcomes the members. She announces that [the user] will be her co-animator for the meeting. Observer’s note: This was only related to the fact that he’s sitting up front; he’s not involved in the moderation at all. However, he will have to give feedback to the [community partner] who is absent.” (Observation notes, CTO meeting - Pathway Physical disability/cerebrovascular accident, May 16, 2018)

The diversity of viewpoints, both from users and cross-sector partners, could also be improved:

“Perhaps having more than one representative per sector is helpful. I’ve attended almost all the meetings, but my colleague from Sherbrooke has missed some. I was impressed by the number of field representatives, and we are as important as they, if not more.” My rural community is very collaborative and full of initiatives. In contrast, my other colleagues come from much further away. The distance from the central city makes it hard to bring people together. Their perspectives might have enriched our discussions. (Respondent #29, partner)

However, this (relative) success at the operational level is not mirrored at the strategic and tactical levels, where the absence of these external stakeholders’ points to a significant gap in organizational coherence. This disconnect hinders the development of a shared vision that integrates the environment into the strategic planning and decision-making processes, a process of coevolution.

The lack of coordinated governance structures that bridge the hierarchical and functional aspects of the organization further exacerbates this issue. There are no established processes or bodies at the strategic and tactical levels dedicated to piloting and liaising with external stakeholders, which poses significant challenges. Firstly, there is a misalignment between the strategic objectives and the deployment of social and health pathways. Without clear communication and integration of external perspectives at these levels, the strategic goals set by the organization may not fully reflect the realities on the ground, leading to suboptimal outcomes. Secondly, the visibility and ownership of pathway management at the operational level suffer from this disconnect. The absence of external input at the higher levels of governance leads to a situation where operational teams may be less aware of or committed to the broader strategic goals of the organization.

Furthermore, the prioritization of recommendations from SHP-OC across different directions and pathways is compromised by the lack of external coherence. The roles of directors, medical managers, and their deputies in aligning these recommendations with the organization’s strategic priorities are unclear, leading to inconsistencies in the implementation process. This lack of coherence is not only a governance issue but also impacts the decision-making processes, particularly concerning the relevance and justification of those decisions.

Finally, the roles and representation of partners and users in decision-making and governance structures are crucial for maintaining external coherence. While there is strong involvement of partners and users at the operational level, their representation is minimal at the strategic and tactical levels. This lack of representation poses a risk of information loss and weakens the coordination of service offerings across territories. Moreover, it undermines the alignment with policies like the Population Responsibility and Community Development,^35,36^ which aim to enhance the participation of users and partners in shaping healthcare services.

In conclusion, achieving external coherence in social and health pathways implementation requires a clear, shared vision of roles and responsibilities across all levels of the organization and its external environment. Without this, the potential for effective, user-centered pathways is significantly diminished, and the organization’s ability to adapt and evolve in response to the needs of its users and partners is compromised.

## Discussion

To discuss the results of our analyses, we have chosen to put them into images (and colors) by taking up the visual of the theoretical framework (Figure 1).

Thus, the following three figures show the successes (in green), the stumbling blocks (in red), and the variations between the two (yellow) of co-evolution at each organizational level and in each clinical and administrative sphere in the implementation of the SHP management in the Healthcare Organization A.

Each figure shows the degree of coevolution for each level of decision making (operational, tactical and strategic) and for each clinical or administrative sphere.

The focus of Research Proposal 1 on internal coherence is illustrated by Figure 3, which highlights coevolution at the top and administrative levels. These levels are GREEN and have seen the development of vision, tools, and methodology. In contrast, the strategic admin level is RED due to barriers that have been erected against the management project from the outset. These barriers include political, symbolic, and functional reasons. The project’s social and health pathways management vision wasn’t clearly shared, leading to a sense of exclusion. The role of the co-owner was poorly defined, resulting in an unproductive project. Several departments felt their leadership was under threat. This explains the silo work, which was not the original goal of transversal governance.

For the other levels in ORANGE, there are variations from case to case, but overall, the more management and teams were involved, the easier it was to commit to and progress with the adoption and implementation of trajectory-based management. This is in line with many organizational studies, but most importantly it supports the fact that management’s confidence in the frontline teams, whether administrative or clinical, is an important marker of success for a project of this scale. Here, the pulse was taken at field level regarding the possible use of trajectory-based management: those responsible decided to go at the pace of each team, rather than in a standardized way as required by some of the literature.^37,38^ This made it possible to contextualize each implementation, to fully understand expectations at all levels and to reconsider certain actions if they were not ultimately on the right track. In short, the implementation was more collaborative than imposed, and this was reflected in the results.

For the research proposal concerning the coherence between the existing structure and the resulting logic of action within the organization, Figure 4 clearly shows the disparities and variations between the decision-making levels and the clinical and administrative spheres.

For the tactical and strategic levels, GREEN represents strong coherence. Coevolutionary principles apply between the different actors: the actions of the tactical levels enable the strategic top to adapt, and vice versa: communication corridors are established, and decisions are made by the right people at the right time.

This is not the case for the strategic level, which is completely in the RED: the issues raised in Figure 3 are major in terms of finding coherence between the structure and the logic of action: although some departments are playing the game, this is not enough to reverse the trend. Both clinical and administrative management are disengaged and respond little or not at all to the requests of the trajectory committees. Stalemate has set in. This is one of the reasons that explain the green light between the other two levels, which had to find ways to act despite everything.

At the operational level, the orange color indicates that the coherence between the structures set up and the logic of action varies. However, operational committees have enabled the implementation of social and health pathways in users’ and patients’ interests.

Co-evolutionary actions between doctors, clinicians, and managers have been implemented (eg, improving adapted transport for people with chronic illnesses): They have learned to work together in a cooperative way and not by thinking that the others know. In short, at this level, communication issues were mostly raised and addressed. This was not the case, for example, for communication issues between hierarchical levels, which limited the operational level’s scope for action.

Research Proposal 3 is shown in Figure 5. The strategic level is red because if the problems exist internally, they are also reflected in the cohesion with the external environment, that is, users, partners, and the community. At the time of data collection, there were no bridges between the strategic levels and the external actors regarding the implementation of social and health pathways.

Conversely, the rest of the organization is committed to continuous improvement, fostering user, and partner involvement through operational (regular) and tactical (by invitation) committees. The top strategic level emphasizes this commitment by addressing participation issues across various institutional bodies. While the implications for coevolution may not always reflect ideal interactions, the established structure significantly facilitates the participation of the organization’s users and partners.

To enhance system performance, it is essential to foster coevolution at all levels – operational, tactical, and strategic – as well as with the external environment. This necessitates the creation of both formal and informal channels of communication, with the objective of ensuring uninterrupted and seamless communication. Furthermore, it is essential to identify key influencers who can effectively disseminate information and interpretations both vertically and horizontally. This approach ensures a seamless transfer of knowledge and enhances coordination among various stakeholders. This process of “setting the scene”^39^ lays the groundwork for robust communication and collaboration, facilitating a well-integrated system.

The acknowledgment of mutual interdependence and the implementation of mechanisms to reinforce this interdependence are of paramount importance for the effective management of the heightened complexity of social and health pathways. The implementation of novel and creative animation principles has the potential to enhance governance by offering more adaptable methodologies aligned with evolving circumstances. However, it is important to note that these developments may also give rise to tensions with traditional governance models that are already in place. This is the case with the strategic level, which, by and large, has not overcome these tensions with the rest of the organization. This has led to misunderstandings and even disconnects between the different decision-making levels.

But these tensions could be, in fact, beneficial as they provide an opportunity to re-examine and adjust vertical and horizontal interdependencies as needed. The incorporation of feedback permits the continuous improvement of the implementation of organizational innovations, such as social and health pathways management.

### Limits

This study has its limitations. Firstly, the results presented predate the COVID-19 pandemic, a period during which structures and players evolved. Despite this, the knowledge acquired during this first study (2017-2019) remains necessary to understanding the history of social and health pathways-based management implementation in Quebec. Secondly, the representation of users was limited to four respondents and that of partners (community, municipal, etc.) to three, which may have biased the analysis in favor of the healthcare sector perspective. Finally, as this study was carried out in a specific region of Quebec, its results may not be directly transferable to other contexts, limiting the scope of the research. Nevertheless, the framework based on complex adaptive systems remains relevant and promising, not only for understanding the internal dynamics of healthcare organizations, but also for exploring co-evolutionary interactions and emerging dynamics with external actors.

## Conclusion

When an organization establishes the need to adapt its services to the needs of the population, it should play a key role in the process of collective adaptation of its governance. The construction of a convergent collective governance system and the emergence of solutions necessitate that these are controlled by the organization’s leaders and are highly animated by its stakeholders.^40^ If we want individual and collective practices to evolve, we need to look at how the organizational schemata work. It is important to put users and the community at the center of the system, not just to focus on services and the medical profession. This means moving from a hospital-centric approach to a more integrated, networked vision where users and the community play an active role in the process. From this perspective, the adaptive governance structure represented by the multi-level coherence of schemata becomes the lever for action.

Schemata are frequently overlooked in change management, with a focus on rational and administrative levers. The development of multi-level governance allows for the refocusing of all actions and schemata to avoid isolated, non-integrated, and non-recursive actions. This facilitates the dynamic process of adapting and changing clinical and organizational practices regarding the populations served by health and social services organizations. The orchestration of multilevel governance within a complex organization allows all heterogeneities to be made homogenous, to converge the various actions targeting adaptation. As the common goal of the organization and its actors is to provide healthcare quality and equity to all users, the development of multi-level governance should enable decisions to be better shared with users and communities.

The coevolutionary principle of social and health pathways in a healthcare system is a strategy for shared decision-making and dynamic involvement for an overall improved performance. To achieve this, it is necessary to maintain a balance and internal coherence between the structure being set up and the existing structure, and to establish formal and informal communication channels to ensure seamless interactions, while recognizing and reinforcing mutual interdependence in a systemic perspective.

## Supplemental Material

sj-doc-1-his-10.1177_11786329251332797 – Supplemental material for Promoting Coevolution Between Healthcare Organizations and Communities as Part of Social and Health Pathways Management in Quebec: Contributions of the Complex Adaptive Systems Approach Supplemental material, sj-doc-1-his-10.1177_11786329251332797 for Promoting Coevolution Between Healthcare Organizations and Communities as Part of Social and Health Pathways Management in Quebec: Contributions of the Complex Adaptive Systems Approach by Lara Maillet, Georges-Charles Thiebaut, Anna Goudet and Jean-Sébastien Marchand in Health Services Insights

## Statements and Declarations

**Data Availability:** The datasets generated and/or analyzed during the current study are not publicly available due to ethics agreements but are available from the corresponding author on reasonable request.
