# Supplementary material for: Promoting Coevolution Between Healthcare Organizations and Communities as Part of Social and Health Pathways Management in Quebec: Contributions of the Complex Adaptive Systems Approach
Source: Health Serv Insights. 2025 Apr 18;18:11786329251332797. doi: 10.1177/11786329251332797 (PMC12033647; doi:10.1177/11786329251332797)
Supplement: sj-doc-1-his-10.1177_11786329251332797 – Supplemental material for Promoting Coevolution Between Healthcare Organizations and Communities as Part of Social and Health Pathways Management in Quebec: Contributions of the Complex Adaptive Systems Approach [file sj-doc-1-his-10.1177_11786329251332797.doc]

**CIUSSS ESTRIE-CHUS Trajectory implementation and animation PROCESS: developmental evalutation (PILOTE #1)**

**CODE: _______________ (1) (linked to ID on separate document)**

**DATE: _______________ (2)**

**INTERVIEWERS: _____________________________________________ (3)**

**IMPORTANT: This questionnaire must not contain any personal or identifying information.**

**Interview with SSSS NETWORK MANAGERS INVOLVED in the development and implementation of trajectory animation at CIUSSS Estrie-CHUS**

**Prerequisites:**

**1. Introducing the interviewers**

*Thank you for agreeing to take part in this research project. Our aim is to study the development and implementation of a trajectory animation project at the CIUSSS Estrie-CHUS. Take as much time as you need to think and answer the questions. There are no right or wrong answers. During the interview, please let us know if you need clarification on any of our questions. We remind you that what you tell us will remain confidential and that the interview will last no longer than 60 minutes. For ethical reasons, I need to get your consent before I start the interview (see consent form).*

**2. Presentation of consent form / answering questions / signing form**

**3. Start of recording**

**DRAFT - GUIDE FOR SSSS NETWORK MANAGERS INVOLVED IN THE TRAJECTORY ANIMATION PROJECT**

| **A) Characteristics of the respondents** | |
| --- | --- |
| - What is your role within the CIUSSS de l'Estrie-CHUS? How do you define your role? - How long have you been working for CIUSSS de l'Estrie-CHUS? |  |

| **B) Respondent's perception of the trajectory animation project** | |
| --- | --- |
| - Could you describe the trajectory animation project? | - What is its purpose? - Does the vision associated with the trajectory animation project (i.e. mission, values, principles) reflect your own vision for the project? - What is your opinion on the compatibility of the trajectory animation project with the values and goals of the organisation? |
| - Could you tell me how and from whom you heard about the trajectory animation project? | - How would you characterise the information you received? - Did you receive any training? What is your perception of this training (adequate, sufficient)? |
| - What is the interest of the Trajectory Animation Project for your service/department/management? |  |
| Specifically in relation to the _________ trajectory,   - What is your role? What do you think about this role? | - What would you change? How could it be improved? |
| - How do you integrate this project into your daily work? | - Do you have any suggestions to encourage your involvement in the trajectory work? |
| - How is the Trajectory animation project perceived in your department? |  |

| **C) Governance in action** | |
| --- | --- |
| **Specifically in relation to the _________ trajectory,**   - Do you think that all the actors involved in the trajectory are present? Please explain. | - To what extent has the absence of certain actors affected the progress of the trajectory? |
| - Can you tell us about the involvement of the actor’s present? | - What is your opinion of the involvement of managers/doctors/community partners/users? - Do you feel that the people involved are ready to join forces and work together? - In general, are you satisfied with the level of collaboration between the different actors involved in the implementation of the trajectories? - How could this collaboration be improved? |
| - What can you tell me about the collaborative climate? | - Do you feel that everyone involved shares the same vision? - If there are differences of opinion, what are they about? What are the convergences? - Are there any dominant opinions? - How difficulties (e.g. disagreements, tensions, conflicts) between people are managed? |
| - How are the other actions related to the implementation of the trajectory animation project coordinated? | - What resources are mobilised to set up the trajectory animation? |
| - How will decisions be taken? | - Was there a formal decision-making process? - What information was provided? - How were divergent opinions considered? |
| - How is your opinion considered in the decision-making process? Can you give an example? | - How did you feel during the decision-making process? |
| **D) Process** | |
| Specifically in relation to the _________ trajectory,   - How do you prepare for these meetings?   What do you do after the meetings? | - What information do you receive? - How is information exchanged between the various bodies involved (C-CAT, core, CAT, GT, etc.)? - Are there any exchanges or preparatory meetings? Who are the people involved? |
| - How did the meetings go? |  |
| - Did you express yourself during these meetings? Could you give us an example? | - Did you feel that other people listened to each participant? |
| - How was your opinion considered in the discussions? Can you share an example? |  |
| - Did you feel that you had an influence on the discussions? Are you satisfied about this? |  |

| **E) Influencing factors (facilitators and barriers)** | |
| --- | --- |
| - In your opinion, have there been any changes in recent years that have affected the work on the trajectory? | Note: If necessary, give an example such as the MSSS reform or orientations. |
| - What factors have made the work easier? |  |
| - What challenges did you face? How were they overcome? |  |

| **F) Perceived effects of project involvement** | |
| --- | --- |
| - What do you learn from your involvement in the project? |  |

| **G) Perceived impact of the trajectory implementation** | |
| --- | --- |
| - Have you observed any effects of the trajectory implementation and animation project:   - On the services organisation?   - On the services offered?   - On the populations reached?   - On the collaboration between the CIUSSS Estrie-CHUS services and their partners? |  |

| **H) Perceived impact on integration** | |
| --- | --- |
| - What has the implementation of trajectory animation changed for your department? |  |
| - Have you noticed any changes in the way decisions are made within the organisation, for example in terms of resource allocation? |  |
| - What can you say about the adaptation of information systems and management methods to the trajectory animation project? |  |
| - In your opinion, is the trajectory animation project well integrated with the rest of the organisation? Please explain. |  |

| **I) Evaluation and perspective** | |
| --- | --- |
| - Overall, how do you assess the development and implementation of the trajectory animation project? |  |
| - To what extent do you feel that trajectory animation is an appropriate solution for improving accessibility and continuity of care and services? - What does accessibility mean for your department? And continuity/fluidity? | - What do you think are the chances of success of such an initiative? - What would you do differently? |
| - Do you plan to continue your involvement in this project? If so, how and why? If not, why not? |  |

| **J) Comments** | |
| --- | --- |
| - Is there anything else you'd like to add to our discussion? |  |
| - Can you provide us with a copy of the documents you mentioned in this interview? |  |

Thank you very much!

**EVALUATING THE PROCESS OF IMPLEMENTING AND FACILITATING THE HEALTH CARE SERVICES TRAJECTORIES AT CIUSSS ESTRIE-CHUS AND MEASURING THE PERFORMANCE OF AN ORGANIZATIONAL AND GOVERNANCE INNOVATION IN 3 CIUSSS’S IN QUEBEC: A MULTISITE DEVELOPMENTAL EVALUATION. (Pilot #2)**

**CODE: _______________ (1) (linked to ID on separate document)**

**DATE: _______________ (2)**

**INTERVIEWERS: _____________________________________________ (3)**

**IMPORTANT: This questionnaire must not contain any personal or identifying information.**

**Interview with MINISTERE DE LA SANTÉ ET DES SERVICES SOCIAUX (MSSS) MEMBERS involved in the development of the trajectory performance measurement and analysis model**

**Prerequisites:**

**1. Introducing the interviewers**

*Thank you for agreeing to take part in this research project. The aim is to study the development of a model for measuring and analyzing trajectory performance. Take as much time as you need to think and answer the questions. There are no right or wrong answers. During the interview, please let us know if you need clarification on any of our questions. We remind you that what you tell us will remain confidential and that the interview will last no longer than 60 minutes. For ethical reasons, I need to get your consent before I start the interview (see consent form).*

**2. Presentation of consent form / answering questions / signing form**

**3. Start of recording**

**DRAFT - GUIDE FOR MINISTRY OF HEALTH AND SOCIAL SERVICES MEMBERS OF THE PROVINCIAL EXECUTIVE COMMITTEE INVOLVED IN THE DEVELOPMENT OF A MODEL FOR TRAJECTORIES MEASUREMENT AND PERFORMANCE**

| **A) Characteristics of the respondents** | |
| --- | --- |
| - How long have you worked for the MSSS? - What is your role within the MSSS? - Can you briefly tell us about your professional background? | - Have you previously held other positions within the MSSS? Or within a CIUSSS (or before 2015 in institutions-CLSC, CSSS, GMF, etc.)? |

| **B) Initial conditions for implementation** | |
| --- | --- |
| - How did you get involved in the Trajectories project? - How and when did you hear about Trajectories and its animation? - What led you to join the provincial committee? | - Could you tell me how and from whom you heard about the trajectory animation project? - How was the project presented to you? How would you describe the information you received? - Did you receive any training? What is your perception of this training (adequate, sufficient)? |
| **Can you tell us how the Trajectories project started?**   - Initial context: both internally and externally to your organisation? - In terms of the organisation's orientations? - In terms of strategic positioning? - Can you tell us how the CIUSSS were selected? | - How would you describe the initial conditions, in other words the context in which the project started? - Who were the actors and what was their role? What was your role? - How did intersectoral collaboration start and develop? |
| **Regarding your role in the development and implementation of trajectory animation:**   - How do you perceive this role? - Does this role have evolved since the start of the project? If so, how? If not, how do you explain why? |  |

| **C) Respondent's perception of the trajectory animation project** | |
| --- | --- |
| - Could you describe the trajectory animation project? - What does **accessibility** mean to your department/direction/service? What about continuity/fluidity? - What does **continuity and fluidity** mean to your department/direction/service? | - What is its purpose? - Does the vision associated with the trajectory animation project (i.e. mission, values, principles) reflect your own vision for the project? - What is your opinion on the compatibility of the trajectory animation project with the values and goals of the organisation? |
| - In your opinion, what are the interests of your service/department/management (CIUSSS) in the trajectory animation project? - And, at the provincial level? | - More specifically, how is this project perceived in your department? |

| **D) Process and governance in action** | |
| --- | --- |
| **In terms of the trajectory animation project implementation process:**   - How are actions coordinated? | - What resources are mobilized to implement the trajectory animation project? |
| **Specifically in relation to the _________ trajectory, or sub-trajectory CATs __________,**   - What activities (formal/informal) do you conduct? | - How do you prepare for these meetings? - What do you do after the meetings? - What about underground activities? |
| **Specifically in relation to the _________ trajectory, or sub-trajectory CATs __________,**   - What can you tell me about the collaborative climate? | - Do you feel that everyone involved shares the same vision? - If there are differences of opinion, what are they about? What are the convergences? - Are there any dominant opinions? - How difficulties (e.g. disagreements, tensions, conflicts) between people are managed? |
| **Specifically in relation to the _________ trajectory, or sub-trajectory CATs __________,**   - Did you notice any changes in actors’ involvement and interest during the project? Could you please explain. | - To what extent did the absence of certain actors have an impact on the progress of the project? - What could be done to improve? |
| **Specifically in relation to the _________ trajectory, or sub-trajectory CATs __________,**   - How will decisions be taken? | - Was there a formal decision-making process? - What information was provided? - How were divergent opinions considered? |
| - How is your opinion considered in the decision-making process? Can you give an example? | - How did you feel during the decision-making process? |
| **Regarding the performance measurement and indicator system:**   - How do you work with this team/instance? | - How do the two teams work together (performance indicator measurement, information exchange)? - Is there a formal or informal process? |

| **E) Influencing factors (facilitators and barriers)** | |
| --- | --- |
| - In your opinion, have there been any changes in recent years that have affected the work on the trajectory? | Note: If necessary, give an example such as the MSSS reform or orientations. |
| - What factors have made the work easier?   About:   - - The Trajectories project development and implementation process?   - The trajectory workflow ____________? |  |
| - What challenges did you face?   - The Trajectories project development and implementation process?   - The trajectory workflow____________? | - How were they overcome? |

| **F) Perceived effects of project involvement** | |
| --- | --- |
| - Have you observed any effects of the trajectory implementation and animation project:   - On the services organisation?   - On the services offered?   - On the populations reached?   - On the collaboration between the CIUSSS and their partners? |  |

| **G) Perceived impact on integration - ONLY FOR FURTHER INFORMATION** | |
| --- | --- |
| - What has the implementation of trajectory animation changed for your service/department/direction? |  |
| - Have you noticed any changes in the way decisions are made within the organisation, for example in terms of resource allocation? |  |
| - What can you say about the adaptation of information systems and management methods to the trajectory animation project? |  |
| - In your opinion, is the trajectory animation project well integrated with the rest of the organisation? Please explain. | - Could you give us some examples? |

| **I) Evaluation and perspective** | |
| --- | --- |
| - In your opinion, what are the main expected/obtained impacts of the trajectory animation project? Why? | - To what extent do you think trajectory animation is an appropriate solution for improving __________? - Could there have been a way to have a greater impact? How could it have been done? |
| - How do you plan to scale up this innovation? |  |
| - To this day, what seems to you to be the most decisive factor for the future of the Trajectories project? | What factors could contribute to:  o the viability of the project? |
| - What do you think are the chances of success of such an initiative? |  |

| **J) Comments** | |
| --- | --- |
| - Is there anything else you'd like to add to our discussion? |  |
| - Can you provide us with a copy of the documents you mentioned in this interview? |  |

Thank you very much !

**EVALUATION OF THE IMPLEMENTATION OF TRAJECTORY MANAGEMENT IN THE HEALTH CARE SYSTEM AND THE VARIATION OF EFFECTS IN CI(U)SSS IN QUEBEC (project #3)**

**CODE: _______________ (1) (linked to ID on separate document)**

**DATE: _______________ (2)**

**INTERVIEWERS: _____________________________________________ (3)**

**IMPORTANT: This questionnaire must not contain any personal or identifying information.**

**Interviews with operational, tactical and/or strategic actors in a CI(U)SSS involved in the implementation of trajectory-based management**

**Prerequisites:**

**1. Introducing the interviewers**

*Thank you for agreeing to take part in this research project. The aim of this research is to study the various factors involved in implementing* trajectory-based management *in different contexts (CI(U)SSS), and then in a potential provincial scaling-up process. Questions will also be asked about the challenges of* trajectory-based management *in the current COVID19 context. Take as much time as you need to think and answer the questions. There are no right or wrong answers. During the interview, please let us know if you need clarification on any of our questions. We remind you that what you tell us will remain confidential and that the interview will last no longer than 60 minutes. For ethical reasons, I need to get your consent before I start the interview (see consent form).*

**2. Presentation of consent form / answering questions / signing form**

**3. Start of recording**

| **A) Characteristics of the respondents** | |
| --- | --- |
| - How long have you worked for the CI(U)SSS? - What is your role within the CI(U)SSS? - Can you briefly tell us about your professional background? | - Have you previously held other positions within the CI(U)SSS? |

| **B) Initial conditions for implementation** | |
| --- | --- |
| - How did you get involved in the trajectory-based management? - How and when did you hear about trajectory-based management? | - Could you tell me how and from whom you heard about the trajectory animation project? - How was the project presented to you? How would you describe the information you received? - Did you receive any training? What is your perception of this training (adequate, sufficient)? |
| - Can you tell us how the trajectory-based management start?   - In the healthcare system? (Québec)   - Initial context: both internally and externally to your organisation? | - How would you describe the initial conditions, in other words the context in which the project started? - Who are the partners and what was their role? How were the partners selected? - What was your role? - How did intersectoral collaboration start and develop? |
| Regarding your role in the development and implementation of trajectory:   - How do you perceive this role? - Does this role have evolved since the start of the project? If so, how? If not, how do you explain why? |  |

| **C) Respondent's perception of the trajectory animation project** | |
| --- | --- |
| - Could you describe the trajectory trajectory-based management in your CI(U)SSS? | - What is its purpose? - Does the vision associated with the trajectory animation project (i.e. mission, values, principles) reflect your own vision for the project? - What is your opinion on the compatibility of the trajectory-based management with the values and goals of the organisation? - What does accessibility mean to your department/direction/service? - What does continuity and fluidity mean to your department/direction/service? |
| - In your opinion, what are the interests of your service/department/management (CIUSSS) in the trajectory-based management project? | - More specifically, how is this project perceived in your department? |

| **D) Process and governance in action** | |
| --- | --- |
| **Specifically in relation to the _________ trajectory.**   - How are decisions taken? | - What activities (formal/informal) do you conduct? - How do you prepare for these meetings? - What do you do after the meetings? - What about underground activities? |
| **Specifically in relation to the _________ trajectory**   - What can you tell me about the collaborative climate? | - Do you feel that everyone involved shares the same vision? - If there are differences of opinion, what are they about? What are the convergences? - Are there any dominant opinions? - How difficulties (e.g. disagreements, tensions, conflicts) between people are managed? |
| - What strategies do you identify for mobilizing CI(U)SSS stakeholders and partners around trajectory-based management in your CI(U)SSS ? | - Who are your partners? What are their characteristics? How were they chosen? - What types of interaction do you have with them? - What are the mechanisms for collaboration trajectory-based management? - What could be done to improve collaborative practices? |
| **Specifically in relation to the _________ trajectory**   - Did you notice any changes in actors’ involvement and interest during the project? Could you please explain. | - To what extent did the absence of certain actors have an impact on the progress of the project? - What could be done to improve? |
| - How is your opinion considered in the decision-making process? Can you give an example? | - How did you feel during the decision-making process? |
| **Regarding the performance measurement and indicator system:**   - How do you work with this team/instance? (Evaluation, Quality, Performance and Ethics Department/Team) | - How do the two teams (your team and Evaluation, Quality, Performance and Ethics Department/Team) work together (performance indicator measurement, information exchange)? - Is there a formal or informal process? |
| **In your opinion, what are the effects of trajectory-based management on governance?** |  |

| **E) Influencing factors (facilitators and barriers)** | |
| --- | --- |
| - In your opinion, have there been any changes in recent years that have affected the work on the trajectory? | Note: If necessary, give an example such as the MSSS reform or orientations. |
| - What factors have made the work easier? |  |
| - What challenges did you face? | - How were they overcome? |

| **F) Perceived effects of project involvement** | |
| --- | --- |
| - Have you observed any effects of the **trajectory-based management** project:   - On the services organisation?   - On the services offered?   - On the populations reached?   - On the collaboration between the CIUSSS and their partners? |  |

| **G) Perceived effects of the impact of the COVID-19 pandemic on the development and implementation of the trajectory-based management / perceived effects of the impact of the trajectory-based management project on the management of the COVID-19 pandemic** | |
| --- | --- |
| - Could you tell us how your service / department / organisation has adapted to COVID-19? |  |
| - Did the COVID19 pandemic change the work around the trajectory-based management (development/implementation/performance measurement)? | - Can you tell me how the trajectories have been affected? (Example: within the youth, mental health, community services trajectory). - Did the trajectory lines change? How? - Have new trajectories been created? Could you explain? - How did the COVID-19 funding allocated to the institutions modify the trajectories?   - At clinical level?   - At administrative level? |
| - Can you tell us how the COVID19 pandemic has changed the implementation of the trajectory-based management within your CI(U)SSS? | - At a continuum level? - At the strategic level? Tactical level? Operational level? With employees? |
| - In contrast, how has the trajectory base-management made it possible to support intervention during the COVID19 pandemic? | - Adaptation of the services provided to the population. - A response to service breakdowns? |
| - Which new practices (clinical, management, decision-making) have emerged in the COVID19 context to maintain/improve care provision? |  |
| - In your opinion, how does trajectory-based management make it possible to navigate in destabilising contexts?   - What are you doing as a clinician/manager/decision-maker to use this tool and maintain care? |  |
| - In your opinion, which structures (departments, services, partnerships) were the most responsive/resilient? |  |
| - In your opinion, is the trajectory-based management well integrated with the rest of the organisation? Please explain. | - Could you give us some examples? |
| - How would you assess the level of implementation of trajectories within your CI(U)SSS? | - What elements enable you to judge the level of maturity of a trajectory and the GTSS? - Which trajectories are:   - Most mature?   - Emerging/developing?   - Why are some trajectories more developed than others? |

| **I) Evaluation and perspective** | |
| --- | --- |
| - In your opinion, what are the main expected/obtained impacts of the trajectory-based management project? Why? | - To what extent do you think trajectory-based management is an appropriate solution for improving __________? - Could there have been a way to have a greater impact? How could it have been done? |
| - How do you plan to scale up this innovation? |  |
| - To this day, what seems to you to be the most decisive factor for the future of this project? | What factors could contribute to:  o the viability of the project? |
| - What do you think are the chances of success of such an initiative? |  |
| - In your opinion, are social health inequalities among the variables to considered or to be considered in the trajectory-based management? |  |
| - In general, when you think about this project, what would you do differently? |  |

| **J) Comments** | |
| --- | --- |
| - Is there anything else you'd like to add to our discussion? |  |
| - Can you provide us with a copy of the documents you mentioned in this interview? |  |

Thank you very much !
